# Evidence for Strong Mutation Bias toward, and Selection against, U Content in SARS-CoV-2: Implications for Vaccine Design

**DOI:** 10.1093/molbev/msaa188

**Published:** 2020-07-20

**Authors:** Alan M Rice, Atahualpa Castillo Morales, Alexander T Ho, Christine Mordstein, Stefanie Mühlhausen, Samir Watson, Laura Cano, Bethan Young, Grzegorz Kudla, Laurence D Hurst

**Affiliations:** 1 The Milner Centre for Evolution, Department of Biology and Biochemistry, University of Bath, Bath, United Kingdom; 2 MRC Human Genetics Unit, Institute for Genetics and Molecular Medicine, The University of Edinburgh, Edinburgh, United Kingdom; 3 Department of Molecular Biology and Genetics, Aarhus University, Aarhus, Denmark

**Keywords:** SARS-CoV-2, mutation equilibrium, selection, synonymous mutations, vaccine design, viral attenuation

## Abstract

Large-scale re-engineering of synonymous sites is a promising strategy to generate vaccines either through synthesis of attenuated viruses or via codon-optimized genes in DNA vaccines. Attenuation typically relies on deoptimization of codon pairs and maximization of CpG dinucleotide frequencies. So as to formulate evolutionarily informed attenuation strategies that aim to force nucleotide usage against the direction favored by selection, here, we examine available whole-genome sequences of SARS-CoV-2 to infer patterns of mutation and selection on synonymous sites. Analysis of mutational profiles indicates a strong mutation bias toward U. In turn, analysis of observed synonymous site composition implicates selection against U. Accounting for dinucleotide effects reinforces this conclusion, observed UU content being a quarter of that expected under neutrality. Possible mechanisms of selection against U mutations include selection for higher expression, for high mRNA stability or lower immunogenicity of viral genes. Consistent with gene-specific selection against CpG dinucleotides, we observe systematic differences of CpG content between SARS-CoV-2 genes. We propose an evolutionarily informed approach to attenuation that, unusually, seeks to increase usage of the already most common synonymous codons. Comparable analysis of H1N1 and Ebola finds that GC3 deviated from neutral equilibrium is not a universal feature, cautioning against generalization of results.

## Introduction

Multiple strategies toward the development of a SARS-CoV-2 vaccine are being pursued (Thanh Le et al. 2020). These include attenuated or inactivated viruses, replicating and nonreplicating viral vectors, proteins, and nucleic acids (reviewed in Thanh Le et al. [2020]). Some of these strategies, notably DNA or RNA vaccines, rely on the expression of viral genes in humans. These and other modes of vaccine development (e.g., to produce high protein titers) might benefit from synonymous site modification (Gustafsson et al. 2004; Coleman et al. 2008; Kudla et al. 2009; Fath et al. 2011; Bentele et al. 2013; Mordstein et al. 2020). Coding sequence optimization methods can be directed to modification of codon usage, codon pair usage, nucleotide and dinucleotide content, and other properties of coding sequences, with the aim of achieving a desired phenotype, such as increased gene expression (Gustafsson et al. 2004; Kudla et al. 2009; Fath et al. 2011; Bentele et al. 2013; Mordstein et al. 2020), improved immunogenicity (Stachyra et al. 2016), or virus attenuation (Coleman et al. 2008).

The DNA and RNA vaccine design methods that might benefit from synonymous site modification fall broadly into two classes: those that aim to detune the live virus (Coleman et al. 2008; Mueller et al. 2010; Manokaran et al. 2019; Cai et al. 2020) and those that aim to enhance expression of individual genes (Stachyra et al. 2014). As with the expression of any transgene, if one viral gene alone is to be expressed in a vector, for example, as part of a DNA vaccine (Stachyra et al. 2014, 2016), then codon optimization of the gene concerned to enable high gene expression may be desirable, not least because such genes can improve immunogenicity (Stachyra et al. 2016), thereby requiring fewer doses (Wang et al. 2006). Such DNA-based vaccines are regarded as relatively safe as no infective form of the virus is required (Khan 2013).

Viral attenuation differs from other coding sequence optimization strategies in that it aims to produce gene sequences with low expression levels, with the assumption that this will lead to the production of intact (or near intact) viruses with low pathogenicity, which can nevertheless induce an immune response in the host (Coleman et al. 2008). Synthesis of a complete attenuated virus with detuned synonymous sites can however result in a virus almost unable to replicate (Coleman et al. 2008) and, as such, a mosaic synthetic virus, with some genes deoptimized some not, can be preferable (Coleman et al. 2008). Attenuation via modification of many synonymous sites has the advantage that any such virus employed as a vaccine will likely need many mutations to acquire wild-type fitness. Such a strategy is thus likely to be robust to virus/vaccine intrahost evolution (Coleman et al. 2008), this being reinforced by the relatively low mutation rate of SARS-CoV-2 (about one mutation every 2 weeks, 26.9 per year; Hill and Rambaut 2020; Nextstrain 2020). Synonymous codon manipulation has thus been proposed as a viable strategy for SARS-CoV-2 attenuation and vaccine production (Kames et al. 2020). A live attenuated codon deoptimized vaccine is being attempted by three groups (as of July 14th Report of World Health Organization 2020). For further consideration of development and safety aspects of SARS-CoV-2 vaccines, see Peeples (2020) and Amanat and Krammer (2020).

Viral attenuation can be achieved by alteration of synonymous sites as a means to modify the pattern of dinucleotides that bridge between successive codons (alias codon pair bias) while retaining the original protein (Karlin et al. 1994; Rima and McFerran 1997; Coleman et al. 2008). This codon pair bias attenuation effect has recently been shown to be largely owing to increased CpG content (Tulloch et al. 2014; Gaunt et al. 2016). This is very likely to relate to the activity of the human zinc antiviral protein (ZAP) as this targets transcripts with high CpG content (Takata et al. 2017; Ficarelli et al. 2019), although it is by no means the only antiviral protein (supplementary table 1, Supplementary Material online). As might be expected, ZAP is under positive selection owing to host–parasite coevolution (Kerns et al. 2008). This activity of ZAP suggests a simple attenuation strategy for SARS-CoV-2, that is, to increase CpG content (Kames et al. 2020), this being consistent with the observed low CpG enrichment of the virus as sequenced in the wild (Xia 2020), also seen in cytoplasmic viruses more generally (Simmonds et al. 2013). UpA is commonly considered alongside CpG not least because both are underrepresented in native human transcripts (Simmonds et al. 2013) and UpA is cleaved by RNAseL (Odon et al. 2019). Similarly, viruses lacking CpG also tend not to have UpA and engineering increased CpG and UpA attenuates viruses (Simmonds et al. 2013; Odon et al. 2019). UpA depletion in SARS-CoV-2 is weaker than CpG depletion (see below).

Although codon pair bias and dinucleotide composition have been commonly discussed in the context of virus attenuation, these are not the only coding sequence modification strategies that can conceivably produce attenuated viruses. Recently, codon bias (Radhakrishnan et al. 2016; Wu et al. 2019; Buschauer et al. 2020), nucleotide composition (Kudla et al. 2006; Mordstein et al. 2020), and RNA structure (Mauger et al. 2019) have (re-)emerged as important interrelated determinants of gene expression in mammalian cells. Additionally, viral nucleotide and dinucleotide composition have a known role in the immunogenicity of nucleic acids via TLR-7 (Diebold et al. 2004). As a result, understanding forces that operate on synonymous site composition, and on nucleotide content more generally, are central to evolutionarily informed vaccine design, and to our understanding of the biology of SARS-CoV-2. As codon optimization is commonly informed by synonymous site usage in the host genome, we here focus on the relationship between synonymous site selection in the virus and attenuation but are cognizant that lessons learnt may also apply to the optimization problem. Specifically, we aim to discern how selection acts on synonymous sites with a view to engineering the virus against the direction favored by selection on the virus.

One means to test for selection, or more generally forces causing a fixation bias, is to identify a difference between predicted equilibrium nucleotide composition (or dinucleotide composition) under a neutral-mutation bias model and the values observed in the wild. To perform such a test one requires data on the relative rates of different classes of mutations (e.g., A→U and G→C) and from these rates per occurrence of the nucleotide calculate the equilibrium position, that is, the nucleotide content at which the rate of gain by mutation from other residues is equal to the rate of mutational loss. One can then compare observed and neutral equilibrium predicted values, with any discrepancy implicating a fixation bias.

Such methods have revealed commonplace deviations from null neutral expectations. For example, bacteria show a common GC→AT mutational bias (Hershberg and Petrov 2010), and hence a deviation from equilibrium in GC rich bacteria (Hildebrand et al. 2010). Similarly, nonequilibrium TA nucleotide skews (Charneski et al. 2011) have been identified. A recent large survey indicated that G+C deviating from neutral equilibria is also common within both prokaryotes and eukaryotes (Long et al. 2018). To derive this conclusion Lynch and colleagues extracted, from mutation accumulation (MA) experiments or parent–offspring sequencing, mutational profiles for numerous species and showed that the observed G+C content, even at codon third sites, was commonly higher than expected given the profile of mutational events (Long et al. 2018). The cause of this is unresolved, although GC biased gene conversion is one possible explanation (Long et al. 2018).

Rapid, accurate, and common sequencing of epidemic and pandemic pathogens provide a rich source of data from which to derive the mutational profile (Hershberg and Petrov 2010; Hildebrand et al. 2010; Charneski et al. 2011). It is possible to ascribe both ancestral and derived states and hence infer the full mononucleotide mutational matrix (a 4×4, 12 parameter matrix of all possible mutations from one state to another) and, with enough mutations, the full dinucleotide matrix (a 16×16, 240 parameter matrix of all possible mutations from one dinucleotide to another). Here, then, we apply this method to SARS-CoV-2.

Under the assumption of selection against CpG (Xia 2020), we predict that observed GC content would be lower than the neutral mutational equilibrium GC content. Under the assumption that synonymous sites are neutrally evolving, we expect the predicted equilibrium distribution of the four nucleotides at 4-fold degenerate sites so be no different to that observed. We find in support of neither hypothesis. Our data suggest, unusually, that the most common third site residue (U) is also the one selected against. Given this, we thus propose the unusual strategy of increasing the usage of the already most highly used residue so as to degrade performance of the virus. Given that prior evidence indicated that selection for reduced CpG content is particular to just immediate early genes (Lin et al. 2020), we also propose a “gene-bespoke” approach (i.e., one tailored to each gene’s characteristics) sensitive to both CpG and putative selection on synonymous site U.

## Results

### SARS-CoV-2 Mutations Are Heavily GC→U Biased

From the 14,599 genomes, we can identify spontaneous mutations. From these, we derive a mutational matrix and from this, we solve for mutational equilibrium. From 1,151 mutations at 4-fold degenerate third sites, we find a heavily GC→AU biased mutational profile (table 1). From this, we deduce that equilibrium GC (termed GC*) should be 17.13% (95% bootstrap estimates 17.09–17.52). The corresponding number is 17.10% on removing six homoplasies. Specifically, we find: U4* = 65.67%; A4* = 17.20; C4* = 13.09%; G4* = 4.04%. The striking bias toward U has been recently commented on and considered to be consistent with APOBEC editing (Di Giorgio et al. 2020; Simmonds 2020).

**Table 1.** The 4×4 Mutational Matrix for 1,151 Mutations at 4-Fold Synonymous Sites (in italics) and from 5,482 Mutations Observed Anywhere in Codons (not italics).

	Derived Allele				
Reference allele		A	U	C	G
	A	—	*0.04878* 0.02204	*0.01626* 0.01722	*0.12927* 0.10067
	U	*0.02454* 0.01753	—	*0.11528* 0.08912	*0.01296* 0.01296
	C	*0.05842* 0.03545	*0.54124* 0.40877	—	*0.01546* 0.00896
	G	*0.23913* 0.12389	*0.52174* 0.18060	*0.05072* 0.02111	—

Note.—Rates are defined as the number of observed changes per incidence of the nucleotide in the reference genome at 4-fold third sites (italics) or in codons. Note that because of different normalizations, the two sets of numbers are not directly comparable in absolute terms.

**Fig. 1.**
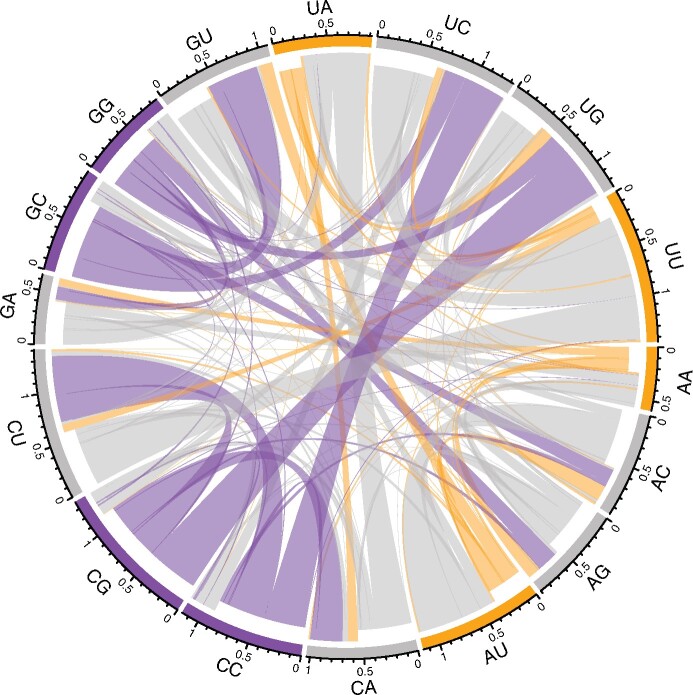
Chord diagram displaying the rate of flux from one dinucleotide to another in the coding sequence of SARS-CoV-2. For each node, the direction of flux is indicated by the indentation of the connecting links: the outermost layer represents flux into the node and the inner layer represents flux out. The frequency of the flux exchange is represented by the width of any given link where it meets the outer axis. Dinucleotide nodes are colored according to their GC-content. Hence, it is evident that there is high flux away from GC-rich dinucleotides whereas AU-rich dinucleotides are largely conserved.

Cognizant that there might be dinucleotide-based mutation biases, we extend the mononucleotide matrix to a 16×16 dinucleotide matrix with 240 parameter estimates derived across the coding sequences (fig. 1 andsupplementary table 6, Supplementary Material online). With 13,209 dinucleotide switches this represents an average of 55.04 mutations per parameter estimate which is liable to be noisy and potentially weakly influenced by selection on nonsynonymous mutations. With this, we determine equilibrium content for all dinucleotides and in turn all nucleotides (A* = 17.66%, C* = 11.65%, U* = 62.42%, G* = 8.27%). We thus estimate from this GC* of 19.92% (95% bootstraps 19.87–20.05%) more or less in line with mononucleotide calculations.

### Evidence for Selection Acting to Counter a Large Mutation Bias toward U

If selection favors reduced G+C content owing to selection for reduced CpG content, we expect that the observed GC3 should be lower than that predicted under neutrality (17.1%). We find the opposite to be true, observed GC3 being 28% (GC3 at 4-fold sites = 20.2%). All numbers are beyond 95% bootstrap bounds of the predicted equilibrium frequency derived from analysis of mononucleotide profiles at 4-fold degenerate sites (bounds: 17.09–17.52). More specifically, at 4-fold synonymous sites, observed U4 (50.8%) is less than predicted under neutral equilibrium U4* (65.7%), whereas all other bases are higher than expected (A4 = 28.95%, A4* = 17.20%; C4 = 13.70%, C4* = 13.09%; G4 = 6.50%, G4* = 4.04%). A parsimonious explanation is that the sizeable mutation bias toward U generates deleterious mutations, nonoptimal even at synonymous sites, and selection therefore favors reduced U content.

**Fig. 2.**
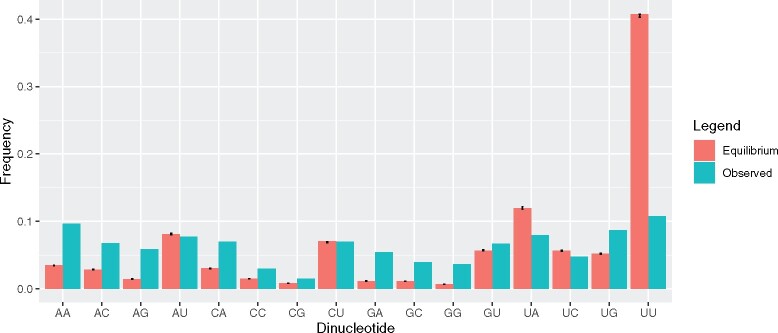
Comparison of dinucleotide content across SARS-CoV-2 compared with neutral expectations. Error bars represent bootstrapped 95% upper and lower confidence bounds.

GC of coding sequence is even more removed from the neutral equilibrium at 38%. This deviation suggests selection in favor of nonsynonymous mutations that increase G+C content. Examination of nonequilibrium status by dinucleotide content supports this. It shows one striking effect, namely that UUs predicted equilibrium frequency greatly exceeds what is observed (fig. 2). More generally, U content whether derived from mononucleotides at 4-fold third sites (predicted 65.7%) or mononucleotides across the genes (predicted 60.3%) or from dinucleotides (62.4%) is greatly in excess of U content, this being 32% for the complete viral sequence. The mutational matrix, whether through mono- or dinucleotide analysis, predicts a great enrichment of U which we infer is being opposed by selection at third sites and in gene bodies (unweighted gene body means: U1% = 25.7%, U2% = 36.3%, U3% = 41%). We notice that CpG content is above that expected under neutrality (fig. 2). However, this we suggest is not so much evidence against selection toward high CpG so much as selection against UU, which by necessity increases the observed relative frequency of CpG and most other dinucleotides as frequencies must sum to one.

### Evidence for Contemporaneous Selection against U at Non-4-Fold Redundant Sites

Possibly consistent with a role for selection, using 5,482 mutations that occur anywhere in the coding sequence (table 1), we observe that the G→U flux at 4-fold degenerate sites is much greater than that observed throughout the sequence. The same is true to a lesser extent for the C→U and A→U fluxes. Assuming the flux rate at 4-fold degenerate sites is more indicative of the true mutational flux, this is consistent with nonsynonymous U mutations being under strong enough selection to be eliminated prior to sequencing. The predicted bias from this matrix is thus slightly more GC rich than that determined from 4-fold redundant sites (GC* at 21.37%: 95% bootstrap estimates 21.39–21.60, 21.44% also after excluding homoplasies).

The lower occurrence of mutations generating U at non-4-fold sites would be consistent with contemporary selection on non-4-fold sites opposing mutations toward U, consistent also with the difference between U4*, U4, and U content overall. To ask whether the difference between the two equilibria solutions is significantly different, we developed a nonparametric Monte Carlo simulation (see Materials and Methods). We find that the Euclidean distances from the random sampling are the same as, or greater than, the Euclidean distance between 4-folds and non-4-fold sites in just 323/10,000 cases (hence *P* = 0.0323) (repeating using an alternative distance metric, sum of modular difference between equilibria, makes no meaningful difference, *P* = 0.0454). To clarify that it was selection against U, we considered each nucleotide individually (see Materials and Methods). Such analysis indeed provides evidence for significant counter selection of U at non-4-fold sites (Un4* = 60.8, U4* = 64.6%, *Z* = −1.98). Commensurably, predicted G equilibrium content derived from mutations at non-4-fold sites is higher than that derived from mutations at 4-fold degenerate sites (*Z* = 5.34), whereas A and C content are less affected (*Z* for A = 0.26, *Z* for C = −0.56). Thus, not only do we detect deviation away from the predicted neutral equilibrium (at 4-fold sites, third sites generally and through the gene body), we also can detect a signal consistent with selection on SARS-CoV-2 that skews the mutational matrix prior to sequencing.

### Significant Heterogeneity in the Degree of CpG Avoidance between Genes

Although selection against U or UU provides a viable model for GC3>GC3*, might there be other explanations that would be consistent with selection against CpG, to avoid ZAP, but in favor of G+C? One possibility is that we may be witnessing between-gene heterogeneity (Digard et al. 2020). Imagine that some genes are indeed under selection for low CpG and hence for low GC3, but others are not under selection for low CpG and thus are more free to have selection favoring higher GC3 (for unspecified reasons, but possibly to enable efficient expression; Mordstein et al. 2020). When then considered en masse, we see both selection for CpG and more raised GC3. Recent reports suggest that not all genes are equally subject to selection for low CpG to avoid ZAP, with only “immediate early” genes under such selection (Lin et al. 2020).

Were this the explanation, or part thereof, we would predict that CpG enrichment would be heterogeneous between genes (see also, Digard et al. [2020]) and that those with relatively high CpG enrichment will also be those genes contributing to raised GC3 (i.e., a positive correlation between CpG enrichment and GC3). Note that, although CpG counts are likely to be necessarily higher as GC3 goes up, CpG enrichment is normalized to underlying GC content and so CpG enrichment and high GC3 are not logically coupled (e.g., if at the limit 50% of residues are C and 50% G, so long as CpG usage is random, CpG = 0.5×0.5, CpG enrichment will not be seen).

**Fig. 3.**
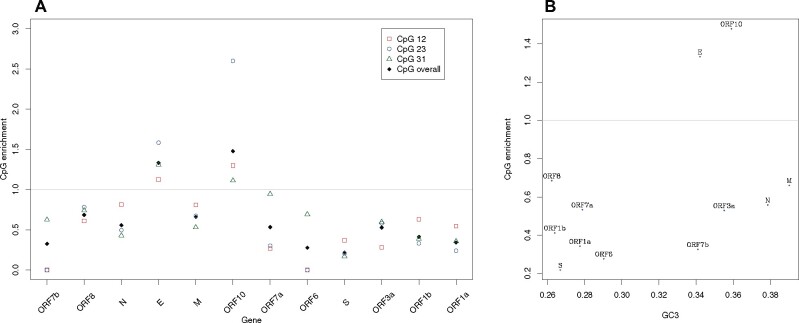
(*a*) CpG enrichment across the genes of SARS-CoV-2. Gray line, no enrichment. (*b*) Relationship between CpG enrichment and GC3.

To assay this, we calculated CpG enrichment at codon sites 12, 23, and 31, these providing three measures of CpG enrichment for each gene. We can then perform a Kruskal–Wallis test (KW) for heterogeneity. Even with such limited data, we find that the three measures for the same gene are more similar than expected by chance (KW, *P* = 0.019, df = 11: mean *E*(CG) = 0.61±0.4 SD; fig. 3*a*). This implies that at all sites CpG is avoided or preferred to the same degree within any given gene. We see however only marginal evidence that genes released from CpG constraint are those with higher GC3 (CpG enrichment vs. GC3, rho = 0.41, *P* = 0.19, Spearman’s test, fig. 3*b*). Thus, although there is evidence for differential CpG usage between genes, we do not find that this predicts GC3, although trends are in the expected direction and the tests underpowered.

**Fig. 4.**
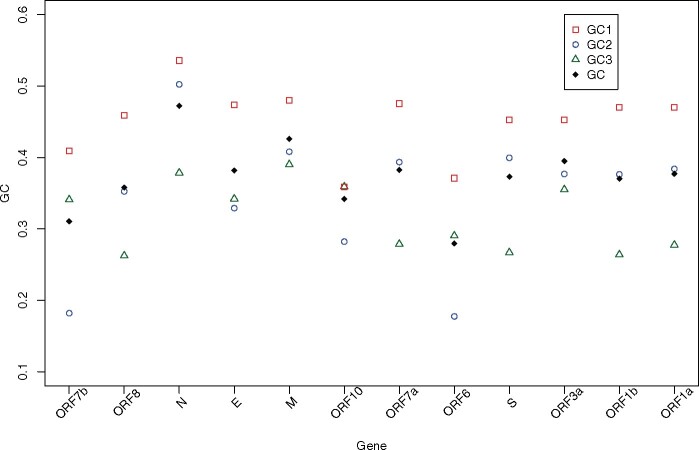
GC content across genes of SARS-CoV-2 at codon sites 1, 2, 3, and averaged across the gene.

More generally, we can ask whether gene body G+C content behaves the same as gene body CpG content with each gene having its own characteristic profile. We assay this by considering GC1, GC2, and GC3 in a manner as above. We find no evidence that genes are more similar in these three measures than expected by chance (KW, *P* = 0.49, df = 11: fig. 4). Similarly, we see no correlation between GC3 and GC12 although the trend is positive (rho = 0.15, *P* = 0.63, Spearman’s rank) (see also, Dilucca et al. [2020]). However, we do observe some regularities. First, GC3 is consistently lower than GC12 (Wilcoxon signed-rank test, *P* = 0.007), the mean GC3 being 28%, whereas that of GC12 is 40%, consistent with selection on amino acid content.

**Fig. 5.**
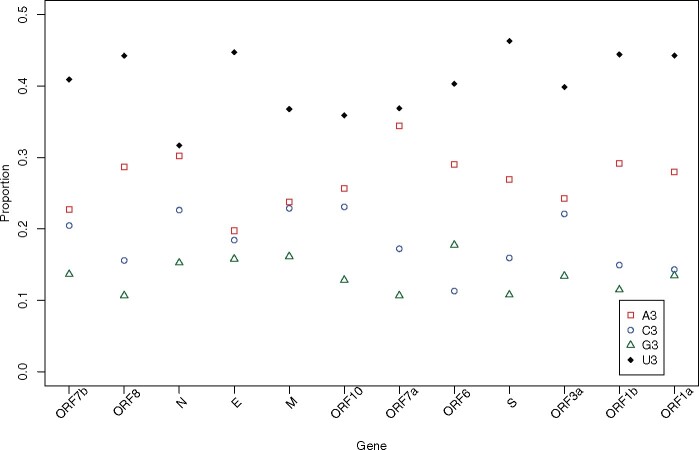
Base composition at codon third sites across genes of SARS-CoV-2.

The most striking feature of third site nucleotide usage is that all genes have a preponderance of U (fig. 5). As noted above, this we can attribute only in some part to mutation as the predicted levels, whereas in the rank order as observed (U>A>C>G) are highly deviant from null. Specifically, the predicted numbers are 0.66 > 0.17 > 0.13 > 0.04, whereas the observed are 0.44 > 0.28 > 0.16 > 0.13. Approximately, the same predicted equilibrium values are seen employing all mutations (0.60 > 0.18 > 0.13 > 0.08). Selection against U seems strong, despite this being the most common nucleotide, as it is heavily reduced from its predicted equilibrium content.

### Genes Avoiding CpG Also Avoid UpA

Prior analysis suggests that viruses lacking CpG also tend not to have UpA and that engineering increased CpG and UpA attenuates viruses, possibly because both are underrepresented in human transcripts (Simmonds et al. 2013). We also observe that UpA enrichment and CpG enrichment tend to positively correlate across viruses (*N* = 1,290, rho = 0.165, *P* = 2.68×10^−9^: data in supplementary table 2, Supplementary Material online). To understanding whether increasing CpG and UpA might be a useful attenuation strategy, we ask whether UpA is also avoided in genes of SARS-CoV-2 and whether it is avoided in the same genes that avoid CpG. We consider not just the CpG enrichment predicting UpA enrichment but also, to control for mononucleotide effects, the two other symmetric nucleotide pairings (ApU and GpC).

**Fig. 6.**
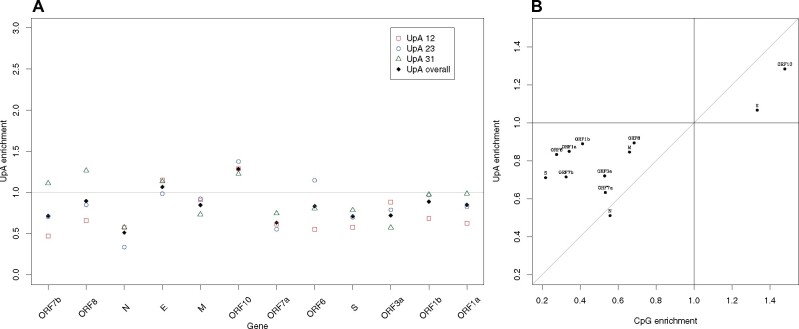
(*a*) UpA enrichment across genes of SARS-CoV-2 and (*b*) correlation with CpG enrichment. Gray line is the line of slope 1 through the origin.

**Fig. 7.**
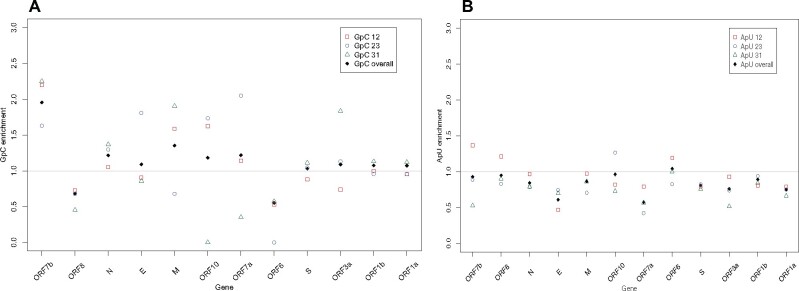
(*a*) GpC and (*b*) ApU enrichment across the genes of SARS-CoV-2.

On an average, UpA is, like CpG, avoided although not to the same extent as CpG (mean UpA enrichment = 0.83±0.2 SD) (fig. 6*b*). UpA also shows between gene heterogeneity (KW, *P* = 0.04). We find that exclusively for CpG enrichment and UpA enrichment do we see a correlation between genes (table 2 and fig. 6*a*). ApU is also avoided (mean enrichment = 0.83±0.14 SD), but there is no evidence for within gene homogeneity (KW test *P* = 0.14) (fig. 7). By contrast, there is no evidence for GpC avoidance: mean GpC enrichment = 1.13, ±0.34 SD (fig. 7) and genes do not show gene-specific GpC enrichment (KW, *P* = 0.11, comparing GpC enrichment at sites 12, 23, and 31). We conclude that if CpG enrichment is a viable strategy to attenuate a gene, increasing UpA may also.

**Table 2.** Between-Gene Correlations in Dinucleotide Enrichment Scores (Pearson product moment correlation *r* values).

	UpAe	ApUe	CpGe	GpCe
UpAe	—	0.20	0.76**	−0.18
ApUe		—	−0.15	−0.16
CpGe			—	0.007
GpCe				—

Note.—Significant correlations in italics:

** *P* < 0.005.

### Evidence for U Content Predicting Expression Level

**Fig. 8.**
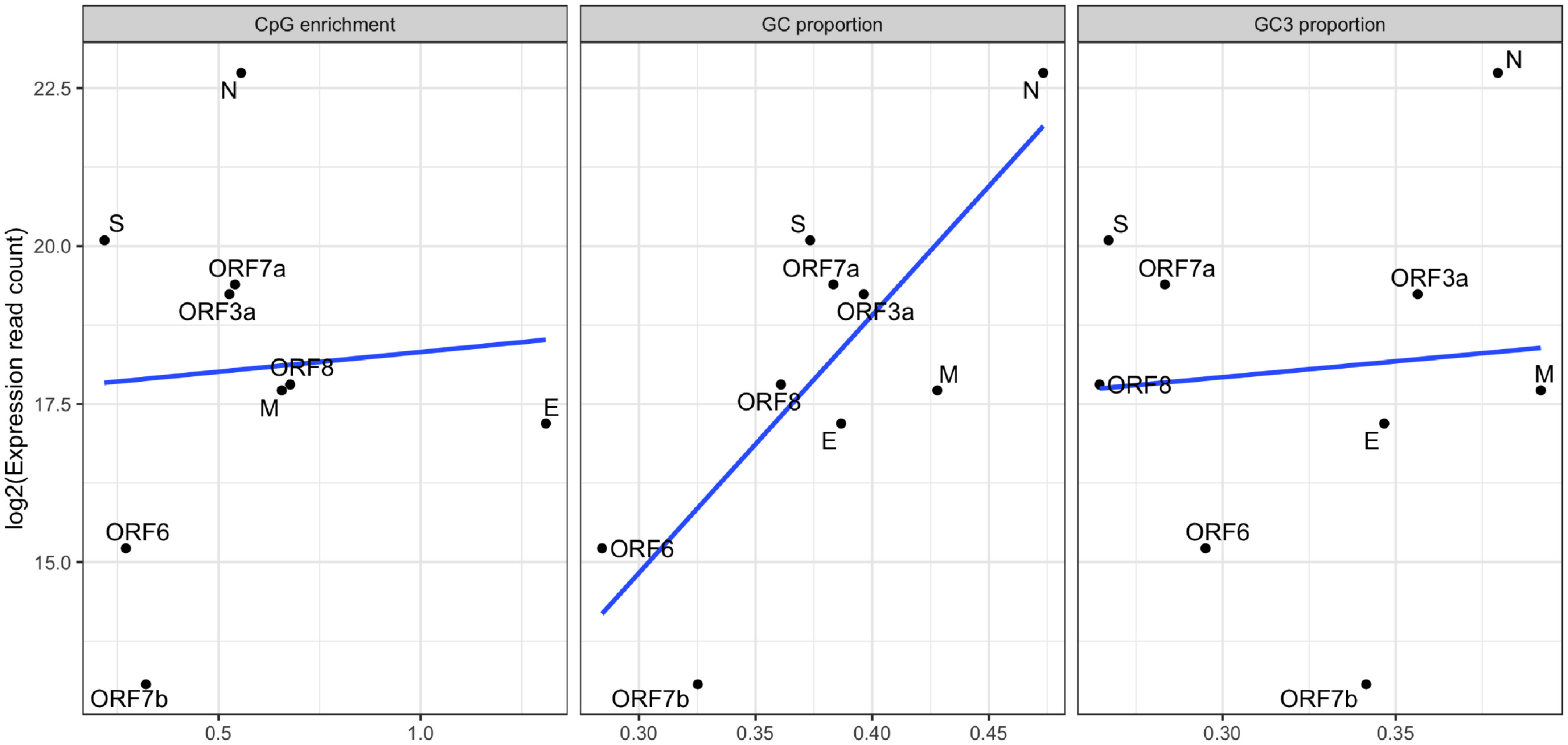
Correlation between expression level and CpG enrichment, GC content, and GC3.

The results above are consistent with a model in which CpG content is under selection in some genes to be reduced, whereas GC3 content is above the level expected under neutrality, in no small part because the U mutation bias is so extreme that equilibrium U content (especially UU content) would render the virus much less fit. There are several possible mechanistic explanations for the GC3>GC3* effect. With our recent evidence that intronless low GC genes are barely expressed in human cell lines (Mordstein et al. 2020), selection for raised GC3 (reduced U3) to enable more effective gene expression is a strong contender. In this context, whereas we do not see a GC3 expression correlation (*r* = 0.09, *P* = 0.82), we do observe a GC expression correlation (*r* = 0.79, *P* = 0.01 and fig. 8). Breaking this down by nucleotide, we see that this is owing to a negative correlation with U content and a positive correlation with both C and G content (A freq: *r* = 0.33, *P* = 0.83; C freq: *r* = 0.64, *P* = 0.06; G freq *r* = 0.81, *P* = 0.009; U freq *r* = −0.88, *P* = 0.0017). Why this is will require considerable experimental manipulation of sequences to understand but we note a correlation between expression level and predicted per nucleotide stability (Pearson’s *r* = −0.86, *P* = 0.0027, df = 7). It is notable that we observe such an effect with such an underpowered test.

**Fig. 9.**
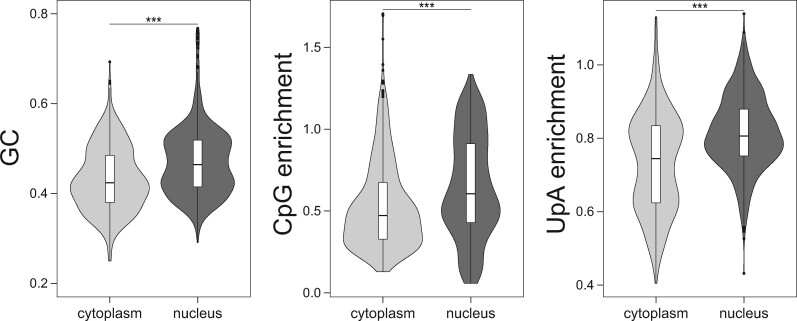
GC content of cytoplasmic and nuclear viruses. Cytoplasmic viruses have significantly lower values for all three measures (Mann–Whitney *U* test: GC: *P* = 6.42e-18, CpG enrichment: *P* = 1.35e-13, UpA enrichment: *P* = 9.1e-29).

A more broad-brush approach is to consider viral sequences more generally (supplementary table 2, Supplementary Material online). As part of the mechanism by which GC enrichment boosts expression is thought to be intranuclear (e.g., nuclear export) (Mordstein et al. 2020), if selection is operating on gene expression of viruses, we might predict that nuclear viruses might have a higher GC content than cytoplasmic viruses. Using mean GC of all viruses within a taxonomic grouping, we observe this to be the case (Mann–Whitney *U* test *P* < 2.2×10^−16^, fig. 9). CpG enrichment and UpA enrichment is similarly lower in cytoplasmic viruses (fig. 9). This is a very arms-length result and requires due caution in its interpretation (it could just as well be evidence of different mutational biases). Nonetheless, within the context of our prior result, we suggest that this merits further scrutiny. There is some evidence that if selection might favor reduced CpG content it might also favor reduced UpA content, as, within both groups, those viruses with low CpG enrichment also tend to have low UpA enrichment, but the effect is weak (Spearman’s test, cytoplasmic viruses: rho = 0.096, *P* = 0.016; nuclear viruses: rho = 0.084, *P* = 0.031).

### Designing the Optimally Attenuated SARS-CoV-2

With the above evidence for selection for G+C at third sites and for heterogeneity between genes in enrichment of CpG and UpA, we suggest that simply increasing CpG by manipulation of synonymous sites need not be the optimal strategy. It may enable recognition by ZAP, but may also favor increased fitness by increasing G+C/reducing U.

As not all genes are under selection for reduced CpG/UpA, reducing their G+C content by increasing U content seems a relatively safe and robust strategy. We thus suggest to classify genes according to the CpG enrichment (>1 or <1). For those in the first category, likely not affected by ZAP (E and ORF10, see also, Digard et al. [2020]), we suggest decreasing their synonymous G+C by increasing where possible U content and forcing them closer to their mutational equilibrium. For those with especially low CpG enrichment and most likely strong targets of ZAP (ORF1a, ORF1b, ORF6, ORF7b, and S), we suggest, raising their CpG, even at the cost of increased G+C. Where possible UpA should also be increased. For the remainder, we suggest increasing CpG content while holding GC3 content static or decreasing if possible. However, with the possibility of synonymous sites also being parts of key motifs, for example, for RNA-binding proteins (Savisaar and Hurst 2017), a simplistic strategy, even if gene-tailored, may have deleterious undesirable side consequences. Unlike alternative strategies that permute existing codons (Jorge et al. 2015), the proposed strategy (supplementary table 7, Supplementary Material online) enables deviations in overall nucleotide content. Recognizing, however, that the extreme nucleotide content can cause gene inactivation, as with prior strategies (Jorge et al. 2015), we propose a stochastic methodology to derive a large number of variants modified in the desired direction that could be experimentally tested (for algorithm, see supplement table 7, Supplementary Material online; for variants for each gene, see supplement table 8, Supplementary Material online). We suggest variants for each gene recognizing the most effective attenuated construct may be a mosaic of wild-type and attenuated genes (Coleman et al. 2008).

### GC3*>GC3 Is Not a General Property of Viruses

We observed that GC content at third sites was both higher than expected given selection against CpG and higher than expected given the underlying mutational profile. Is a deviation from mutational equilibrium a general property of human viruses? Were this so, this too could have implications for engineering of attenuated forms. To address this, we consider other viruses with rich sequencing from epidemics.

For H1N1 using the same mode of analysis, we observe both a less extreme GC→AT mutation bias (table 3) and an observed GC3 content very close to that predicted. From analysis of third sites, the predicted value is GC3* = 41.8% (bootstrap 95% intervals 41.45–42.04), from all sites the prediction is GC* = 42.8% (bootstrap 95% 42.56–42.96). The observed GC3 is 41.8%, within the bounds of the prediction based on third site mutations. For Ebola (table 4), we find observed GC3 is all but identical to predicted (observed GC3 = 46.4%, expected = 46.7%). We conclude that analysis of SARS-CoV-2 and its nonmutational equilibrium status at synonymous sites does not necessarily hold lessons for other viruses. In contrast to others (Kames et al. 2020), we suggest caution in generalizing vaccine strategies.

**Table 3.** The 4×4 Mutational Matrix for 1,522 Mutations at Synonymous Sites (in italics) and from 2,571 Mutations Observed Anywhere in Codons (not italics) for H1N1.

	Derived Allele				
Reference Allele		A	U	C	G
	A	—	*0.0871* 0.04597	*0.065* 0.0451	*0.4291* 0.25542
	U	*0.0803* 0.05143	—	*0.4945* 0.24889	*0.0529* 0.03429
	C	*0.1691* 0.11426	*0.5699* 0.30675	—	*0.0251* 0.02607
	G	*0.6089* 0.32052	*0.0948* 0.05027	*0.0323* 0.0207	—

Note.—Rates are defined as the number of observed changes per incidence of the nucleotide in the reference genome at third sites (italics) or in codons.

**Table 4.** The 4×4 Mutational Matrix for 1,682 Mutations at Synonymous Sites (in italics) and from 3,523 Mutations Observed Anywhere in Codons (not italics) for Ebola.

	Derived Allele				
Reference Allele		A	U	C	G
	A	—	*0.0739* 0.05077	*0.0964* 0.06722	*0.2123* 0.14803
	U	*0.0594* 0.05152	—	*0.2145* 0.13429	*0.0536* 0.04786
	C	*0.0845* 0.08086	*0.2639* 0.14868	—	*0.0394* 0.04845
	G	*0.2639* 0.16051	*0.0751* 0.05139	*0.0694* 0.05139	—

Note.—Rates are defined as the number of observed changes per incidence of the nucleotide in the reference genome at third sites (italics) or in codons.

## Discussion

Mutation bias across all taxa is typically GC→AT biased (Hershberg and Petrov 2010; Hildebrand et al. 2010; Liu et al. 2018) and neutral predicted equilibrium frequencies below GC of 20% (as observed here) are not without precedent (see, e.g., Long et al. [2018]). Broadly the U enrichment at third sites within the genome is then compatible with a large role for mutation bias, possibly mediated by members of the APOBEC gene family (Di Giorgio et al. 2020; Simmonds 2020), known mutators of viruses (Lee et al. 2008) with C→U and UC→UU preferences (Chen and MacCarthy 2017). However, we have shown that nucleotide usage, although skewed in the direction imposed by mutation bias, is nonetheless deviant from it. The difference between observed and expected U3 and UU (fig. 2) proportions are noteworthy. At 4-fold degenerate sites, although C and G usage are close to equilibrium, A is far above and U is far below (U4 = 50.8%, U4* = 65.67%; A4 = 28.95%, A4* = 17.20; C4 = 13.70%, C4* = 13.09; G4 = 6.50%, G4* = 4.04%). We propose that a parsimonious explanation is that the sizeable mutation bias toward U generates deleterious mutations, even at synonymous sites, and selection therefore favors reduced U content. However, increasing C or G potentially comes at a cost of increased CpG, so the base most in excess of its equilibrium is A. As a consequence, although CpG avoidance is real in some genes, GC3 is a little higher than predicted from the underlying mutational profile. This thus presents an unusual case in which the most common synonymous codons (those ending in U) are not the selectively advantageous ones.

We have not directly addressed the problem of the causes of any such selection on synonymous mutations. Given G+C preference in human coding genes to enable effective expression (Kudla et al. 2006; Mordstein et al. 2020), the negative correlation between U usage and expression is broadly consistent with evidence for preferential degradation of transcripts with nonoptimal codon usage (Radhakrishnan et al. 2016; Buschauer et al. 2020). Potentially in tandem to such possible effects high U content may trigger immunogenicity of nucleic acids via TLR-7 (Diebold et al. 2004). Whether a virus with a few more U residues is importantly more immunogenic is, however, uncertain. Alternatively, effects may be mediated by changes in mRNA secondary structure (Mauger et al. 2019). We indeed observe a correlation between expression level and predicted per nucleotide stability. Given this, it could be speculated that RNA stability may explain the thermal intolerance of the virus (Demongeot et al. 2020), although many other mechanisms are imaginable.

On a related note, aside from the possible influence of APOBEC generating the excess of UU mutations, we have not considered the causes of the very different rates for each class of mutation. Indeed, for the high G→U rate, we know of no editing process that has this profile (see supplementary table 1, Supplementary Material online). However, we speculate that this might be owing to oxidation of guanosine that can lead to a G to U transversion. This process may be accelerated by NO via its oxidate species ONOO^−^ (Yermilov et al. 1995; Juedes and Wogan 1996), the former being produced primarily by inducible NO synthase. This enzyme is upregulated in many cells including inflammatory phagocytic cells including macrophages, mediated by proinflammatory cytokines including IFNγ (Zhuang et al. 1998). This has known effects on viral mutation (for review, see Akaike and Maeda [2000]). It may also be informative to consider the relationship between RNA secondary structure and these mutation biases (Krishnan et al. 2004), although overall proportion of variable 4-fold redundant sites does not covary with stability (Pearson’s *r* = −0.06, *P* = 0.88, df = 7).

There are, however, at least four problems with our mode of analysis. First, a theoretical alternative explanation for the difference between predicted and observed values is that the virus was at neutral mutational equilibrium in its prior host (cf. H1N1, Ebola), but since the transfer to humans, the mutational profile has altered. Were this so we may just have identified a lag in viral evolution from one neutral equilibrium to another. In this context, deviation from equilibrium has little if anything to say about either selection or optimal vaccine design. Although evidence for GC→AT biased mutation in related viruses (Simmonds 2020) renders this less parsimonious an explanation, direct examination of mutational profiles of the virus in its ancestral host (whatever that may be) would be valuable. The evidence for subtly but significantly different mutational matrices dependent on the class of site employed provides more direct evidence for contemporary selection on U content throughout gene bodies that cannot be accounted for by a temporal shift in mutational profile.

Second, assuming no change to the mutational matrix, sensu stricto, we have observed a force that would cause a fixation bias (Lercher et al. 2002). Evidence for such a force need not necessarily indicate the direction of selection, as selection bias is only one class of fixation bias. In biased gene conversion, for example, the mismatch repair machinery recognizes, during double-strand break repair, heteroduplex GC:AT mismatches and corrects these in favor of GC residues (Brown and Jiricny 1988). This causes a meiotic drive like process in which deleterious mutations can be driven to higher frequencies (for further consideration, see Hurst [2019]). Given that single-strand RNA cytoplasmic viruses, such as SARS-CoV-2, are unlikely to be exposed to the nuclear mismatch repair machinery or need double-strand break repair, biased gene conversion is unlikely to explain GC3>GC3* and U3<U3*. We cannot with our data, however, rule out unknown mechanisms causing similar nonselective fixation biases. It is then valuable to provide more direct evidence for an advantageous effect of reduced U3/increased GC3, as suggested by our preliminary analysis on expression level. Experimental manipulation of GC3 content (cf. Kudla et al. 2006; Mordstein et al. 2020) is a high priority.

Third, we have presumed that the mutational spectrum observed at 4-fold degenerate sites is a good reflection of the true mutational profile. Often when applying methodology like this, we presume that the temporal proximity between occurrence and observation of mutations is so small that there has been no time for selection to filter in a manner that distorts the mutational matrix. Nonetheless, we found that although slight, there is a difference between the mutational profile observed at CDS sites that are not 4-fold degenerate and those that are. Although this difference is so slight it cannot explain why U is so deviant from equilibrium levels, and does not question our overall findings, we do nonetheless presume that the 4-fold site matrix itself is unbiased. For strains sequenced hours to days apart to be biased at 4-fold degenerate sites would require strong and biased selection at 4-fold redundant sites. Although not obviously plausible, we have no means to disprove this (and strong selection, albeit associated with splicing, has been identified at synonymous sites in human genes; Savisaar and Hurst 2018). Nonetheless, any such bias would also force the mutational matrix observed to predict a nucleotide content more closely resembling the observed nucleotide content, rendering the test conservative. Derivation of the mutational profile by in vivo analysis (cf. Denison et al. 2011) could enable more direct tests of our findings. Analysis of SARS-CoV (responsible for the 2002 SARS outbreak) with exonuclease activity (which we presume to mimic SARS-CoV-2, it having a nsp14 homolog of the SARS-CoV exonuclease; Pachetti et al. 2020) reports a massive AU→GC bias with eight of 11 reported mutations being in this direction and only two GC→AU (Smith et al. 2013). This implies either a radically different mutation bias in SARS-CoV than in SARS-CoV-2 or great sensitivity of results to experimental conditions, such as cell lines employed and APOBEC activity. We note that the SARS-CoV analysis employed Vero cells in which the interferon response is disabled (Smith et al. 2013), thus likely to have neither ZAP (see, e.g., MacDonald et al. [2007]) nor APOBEC activity (see, e.g., Peng et al. [2006]).

Fourth, we have presumed that, after filtering (see Materials and Methods), all sequences are error free. Although sequencing errors cannot explain a bias as strong as the difference between excess and expected UU or U3, nor can they obviously explain the evidence for contemporary selection against U, it may possibly explain the small difference between predicted and observed nucleotide content at 4-fold sites for G and C (the deviations of A and U from predicted equilibria are relatively large). One suggested means to avoid this is to only employ mutations that have been sequenced more than once (Hildebrand et al. 2010). However, this has been shown to introduce its own bias (Charneski et al. 2011). Using high-quality sequence, it was shown that using mutations that appear once and those that appear twice or more makes a significant difference to the matrix and estimates of equilibria (Charneski et al. 2011). The cause of this is likely to be a selection filter: mutations that persist longer to be sequenced twice or more will be skewed toward milder effect mutations. This accords with our observation of a slight difference between matrices that restrict just to 4-fold degenerate sites and those that do not. The ideal then is to filter not by regularity of appearance but by sequencing quality (hence our decisions on which sequences to employ: see Materials and Methods). Nonetheless, to err on the side of caution, we considered mutations at 4-fold degenerate third sites that appear more than once (i.e., excluding singletons) and found that GC* is now even lower than previously predicted (GC* = 10.3%, 95% bounds 10.19–10.61). Thus, we are confident that we can exclude sequencing error as an explanation for observed GC3>GC3* and U4<U4* (singleton excluded prediction of U4* = 65.7%). Nonetheless, owing to observation bias and low sample size, we caution against overinterpretation of this result. Given possible biases owing to sequencing platform, we also ask about the expected equilibrium content for Illumina and Nanopore sequencing separately. We find that the predicted equilibrium vectors for 4-fold degenerate sites are no different from each other (*P* = 0.62).

Assuming we have identified the direction of selection (against U, against CpG in some genes) this can inform vaccine design. Unusually, even though U is the most common nucleotide at third sites (by a considerable margin), we propose increasing this even more thereby forcing the viruses against the direction of purifying selection. We predict that raising CpG in the genes that are CpG deficient would be a viable strategy even at a cost of raising GC3/lowering U. By contrast for those few genes with E(CpG) >1 (i.e., gene E, ORF10, see also, Digard et al. [2020]) CpG manipulation increasing GC3 would be a dangerous strategy, potentially achieving little more than an increase in expression. Increasing their U content would appear to be the antiselection direction. We note however that ORF10s function, if any, remains unclear there being no evidence of transcripts from it, despite it looking like a well-formed ORF (starts ATG stops TAG, multiple of three long). Its GC3 content is also far from neutral equilibrium (GC3 = 36%). In this context, gene E may be a good one to alter synonymous site usage as it appears not to be under selection for CpG or UpA avoidance.

Genes ORF1a, ORF1b, ORF6, ORF7b, and S are good candidates for the raising of CpG content. Gene N is noteworthy in being very highly expressed, long (1,260 bp), GC rich (GC3 = 38%), and with moderate CpG enrichment (E(CpG) = 0.56). Given these characteristics it should be possible to increase CpG by manipulating some third sites (those with C at codon position 2 or G at codon position +1) while reducing GC and increasing U content at other sites. For smaller genes, there is less leeway. In this context S, ORF1a and ORF1b are also very strong candidates being long, with moderate GC3 and low CpG enrichment. A more detailed description of the algorithm for attenuation alongside attenuated variants can be found in supplementary tables 7 and 8, Supplementary Material online. Although the particular strategy for attenuation reflects the particulars of selection operating on SARS-CoV-2, the more general notion of evolutionarily informed vaccine design, with attenuation achieved by synthesizing variants rich in the compositional features opposed by selection, is worthy of experimental scrutiny.

## Materials and Methods

### Gene Locations

We employed NC_045512 to specify the gene sequence to determine observed GC content and CpG content. However, following further annotation of genes (Kim et al. 2020), we modified the gene locations to reflect those specified: https://github.com/hyeshik/sars-cov-2-transcriptome/blob/master/reference/SARS-CoV-2-annotations.gff. Specifically, to avoid a small codon overlap, we exclude the overlap hence employed annotation:

ORF7a protein 27394.27759→27394.27753

ORF7b protein 27756.27887→27762.27887

To consider ORF1a and ORF1b independently and to avoid overlap, we employ:

ORF1a→266-13465

ORF1b→13471-21552

### Estimating Flux Rates from Data

As with parent–offspring sequencing and MA lines, to estimate neutral equilibrium nucleotide content, we require that the mutations observed are an unbiased sample of the mutational profile (Hildebrand et al. 2010; Long et al. 2018). With very common sequencing (in all cases, short-time periods between ancestor and progeny), we can ignore the possibility of multiple sequential hits at the same site (with the first hits going unsequenced) contaminating the mutational matrix. In principle, the method can be misled by strong selection purging, in a nonrandom fashion, mutations prior to their appearing in the population. However, if most selection is weak purifying selection there is then a lag between a deleterious mutation appearing (and being sequenced) and it being purged from a population. Declines in Ka/Ks as time to common ancestry increases in closely related bacteria strains (Rocha et al. 2006) is consistent with such a model. In principle, even if there is strong selection on some mutations this too need not be problematic, so long as strong selection only affects the observed rate of appearance in sequencing data of new mutations but not the relative proportions of the different mutational classes (C→G, A→U, etc.). Moreover, if selection does act in a biased manner it should force the predicted equilibrium to more closely resemble the observed nucleotide content, rendering the test conservative. To be cautious, however, we focus on segregating mutations at 4-fold degenerate synonymous sites as the closest approximation to the underlying mutational profile.

In total, 15,721 SARS-CoV-2 genome assemblies available on 12 May, 2020 were downloaded from the GISAID (Shu and McCauley 2017) Initiative EpiCoV platform. Only assemblies flagged as “complete (>29,000 bp),” “high coverage only,” and from a human isolate were downloaded. Isolates with >1% of ambiguous base calls (rounded to 298 bases) were removed, leaving 14,855 genomes. Sequences were aligned with MAFFT 7.458 (Katoh and Standley 2013) to Wuhan-Hu-1 reference genome (EPI_ISL_402124). EPI_ISL_402124 was collected from a retailer at Huanan Seafood Wholesale Market, Wuhan on December 30, 2019. We employed this sequence as not only was it an early sequence but it also matches the consensus generated from all the 19 sequences that were collected prior to December 31. Variant sites were obtained from the MSA using the package SNP-sites (Page et al. 2016) and whole-genome nucleotide flux estimates were obtained by counting the frequency of each type of mutation with respect to the reference genome. Each given mutation at any given site was counted once, regardless of its frequency within the population. Our method should be insensitive to the presence of recombination, not that there is any evidence that SARS-CoV-2 has recombined through its pandemic phase (Wang et al. 2020). For consideration of homoplasies (independent mutations at the same site), see below.

Isolates containing at least one coding sequence of length not divisible by three were excluded, removing 58 strains, resulting in a set of 14,599 sequences. CDSs were then translated using BioPython, realigned using MAFFT, and then reversed translated using TranslatorX (Abascal et al. 2010). MSA of CDSs were concatenated and then, just as with the whole-genome analysis, variant sites were obtained using SNP-sites and flux estimates were obtained by counting the frequency of each type of change with respect to the reference.

Additionally, H1N1 influenza A pdm09 sequences for strains collected between January 2009 and August 2010 that contained segments PB2, PB1, PA, HA, NP, NA, MP, and NS were obtained from GISAID (Shu and McCauley 2017) for four segments: RNA polymerase subunit (PB2), hemagglutinin (HA), nucleoprotein (NP), and neuraminidase (NA). Sequences with length not divisible by three or containing a stop codon when translated were excluded. Remaining sequences were translated by BioPython and aligned to Mexican strain EPI_ISL_66702 using MAFFT, and reverse translated to nucleotides using TranslatorX (Abascal et al. 2010).

Multiple sequence alignment of 1,610 full Ebola virus (EBOV) genomes sampled between March 17, 2014 and October 24, 2015 in West Africa was downloaded from EbolaID database (Carneiro and Pereira 2016). The alignment includes the reference genome NC_002549.1. Genomes with a proportion of >10% missing sites were discarded. CDSs for each strain were obtained by extracting the coordinates from the reference genome on the alignment. In order to include in the analysis as the largest proportion of the gene ZEBOVgp4, the longest CDS (NP_066246.1) was used, and the shorter, overlapping proteins NP_066247.1 and NP_066248.1 were discarded. Just as in the case of H1N1, sequences with length not divisible by three were excluded. Remaining sequences were translated aligned to the reference strain using MAFFT, and reverse translated to nucleotides using TranslatorX (Abascal et al. 2010).

### Estimating Equilibria

In principle, one can estimate neutral GC equilibria knowing relative rates of GC→AT and AT→GC mutations alone (Long et al. 2018). However, we take a fuller approach to estimate the equilibrium content of all nucleotides that also enables us to capture nucleotides skews (Charneski et al. 2011). This has the advantage of treating all four bases as separate independent states, as is fitting for a single-stranded virus unconstrained by Chargaff’s first parity rule (Elson and Chargaff 1952). Let us denote the frequency of G as *G* and the frequency of U and *U*. We shall write that the mutational frequency of G to U will be g2u, these being measured per occurrence of the starting base. The frequency of the nucleotides after some period (*N*′) will then be:

$$G^{\prime }=G\;\left(1-g2u-g2c-g2a\right)+A\;\left(a2g\right)+U\;\left(u2g\right)+C\;\left(c2g\right)$$

$$C^{\prime }=C\;\left(1-c2u-c2g-c2a\right)+A\;\left(a2c\right)+U\;\left(u2c\right)+G\;\left(g2c\right)$$

$$A^{\prime }=A\;\left(1-a2u-a2c-a2g\right)+G\;\left(g2a\right)+U\;\left(u2a\right)+C\;\left(c2a\right)$$

$$U^{\prime }=U\;\left(1-u2g-u2c-u2a\right)+A\;\left(a2u\right)+G\;\left(g2u\right)+C\;\left(c2u\right).$$

We then solve such that *G*′ = *G* and *U*′ = *U*. This thus resolves to:

G (g2u+g2c+g2a)=A (a2g)+U (u2g)+C (c2g)

C (c2u+c2g+c2a)=A (a2c)+U (u2c)+G (g2c)

A (a2u+a2c+a2g)=G (g2a)+U (u2a)+C (c2a)

U (u2g+u2c+u2a)=A (a2u)+G (g2u)+C (c2u).

Note that the left hand of each equation is the rate of loss given current abundance, whereas the right is the rate of gain given current abundances (i.e., we are solving for gain = loss). The 12 flux parameters (a2u and a2c) we derive from the mutational profile these being the number of observed changes per relevant occurrence of the nucleotide in the ancestral (premutated) sequence. We then solve these four simultaneous equations. Note that, we replace any one arbitrarily chosen frequency by 1−sum of the other three (e.g., *U* = 1−*A*−*C*−*G*). These were solved in NumPy. Equilibrium solutions we denote with an asterisk (e.g., G* and GC3*). N4* implies nucleotide content of nucleotide *N* at 4-fold degenerate sites.

To assign bounds on the equilibrium estimates, we perform a bootstrap test in which we resample with replacement *M* mutations from the set of *M* mutations. For each sampled vector, we recalculate the predicted equilibria thereby assigning bounds. We report 95% bootstrap bounds from 100 resamplings.

The same approach applies to the 16×16 dinucleotide matrix with 240 parameters.

### Comparing Mutational Matrices

We sought to test whether the predicted equilibria solutions were different between the matrices reflecting mutational profiles at 4-fold degenerate sites and all mutations at other sites (i.e., not 4-fold degenerate), as might be predicted were there contemporaneous selection against mutations that are nonsynonymous. We partitioned all CDS mutations into those at 4-fold redundant sites (*n* = 1,151) and all others (*n* = 5,482). Using these two data sets, we calculated observed equilibrium frequencies for each nucleotide (4* for 4-folds and n4* for non-4-folds), representing each as a vector of length four. We then determined the Euclidean distance between the two vectors. To test for significance, we compare the magnitude of this Euclidean distance to that expected by chance employing a nonparametric Monte Carlo simulation. To this end, we randomly extracted without replacement 1,151 mutations from the full set of mutations so as to create a subsample of pseudo “4-folds.” The remaining 5,482 mutations we then considered a sample of pseudo “non-4-fold” mutations. For each randomization, we assembled the corresponding mutational matrix, solved for equilibria, and calculated the Euclidean distance between the resulting vectors of predicted equilibrium for the four nucleotides. We repeated this procedure 10,000 times to generate a null distribution of Euclidean distances that controls for sample sizes differences. Significance was given as *P* = *n/m*, where *n* is the number of simulations in which the Euclidean distance is as great or greater than observed in the real data and *m* is the number of simulations (i.e., 10,000). To check for robustness, we considered an alternative distance metric, namely sum of modular differences (Euclidean distance considers square root of sum of squares of difference).

To consider each nucleotide individually, from the same Monte Carlo sampling, we calculated the difference between predicted equilibria at sampled pseudo “4-folds” and pseudo “non-4-folds” for the 10,000 repeats. This generates four distributions, one for each nucleotide. For each nucleotide, we calculate the mean (∼0) and SD of these randomizations. The observed difference seen for each nucleotide between the equilibria predicted using mutations at 4-fold sites (their predicted neutral equilibria) compared with that calculated using mutations at non-4-fold site, may then be represented as a *Z* score (*Z* = (observed−mean of simulations)/SD of simulations), *Z* > |1.96| indicating significant deviation.

### Homoplasy Screen in SARS-CoV-2

Sites can appear as having independently occurring mutations for at least two reasons: the extra mutation may be a sequencing error or it may be a true homoplasy (i.e., the same mutation at the same site occurring more than once independently) (van Dorp et al. 2020). Sequencing errors need to be removed. Knowing how to handle true homoplasies in the construction of a mutational matrix is not as conceptually simple.

At first sight one might suggest that, as independent mutations, each occurrence of the mutation should be considered. The key question, however, is whether the mutational profile at these sites is representative of activity at other sites. If it is not, then their over inclusion will bias the matrix toward the profile of homoplasic sites away from that of the rest of the genome, which could itself cause a false signal of nonequilibrium status (i.e., where mutationally predicted and observed nucleotide compositions—largely at nonhomoplasic sites—disagree). A priori by virtue of the fact that they are homoplasic we might suppose that mutational activity at these sites is not reflective of the mutational profile elsewhere in the genome and it is the equilibrium properties of other sites that we are interested in. Equally, these may well be sites that are more likely to be under selection (van Dorp et al. 2020) and hence, again, not necessarily reflective of the mutational process. One could then opt to filter out mutations at homoplasic sites considering them possibly unrepresentative. However, we do not know they are unrepresentative and so their removal may be depleting the analysis of information. We also do not know how many of the nonmutated sites had had the property of being homoplasic prior to current sequencing. An alternative, the middle way, is to include them but count all occurrences at any given site as one event, thereby employing the mutations but preventing such sites from overly skewing the matrix and further reducing the impact of possible (missed) sequencing errors.

For analysis of SARS-CoV-2, we opt for the latter “middle way” approach but also check for resilience by removing such sites. Fortunately, as such sites are so rare (6 of 1,151 4-fold degenerate sites), removal of these sites makes no important difference to calculation of GC equilibrium content, nor to estimation of observed nucleotide content. We thus report the homoplasic-excluded results as minor asides.

Phylogenetic tree of 11,204 SARS-CoV-2 isolates was downloaded from the COVID-19 Genomics UK Consortium website (https://www.cogconsortium.uk/, version of April 24, 2020). Subsequently, the MSA and the resulting tree were used to identify recurrent mutations (homoplasies) using HomoplasyFinder (Crispell et al. 2019). All ambiguous sites in the alignment were set to “*N*.” Sites in the first and last 200 bp of the genome alignment were masked to account for the fact that a higher degree of spurious variants that can appear homoplasic tend to locate at the ends of the multiple sequence alignment.

HomoplasyFinder identified 408 putative homoplasies that were distributed over the SARS-CoV-2 genome. Homoplasies can occur as a result of convergent evolution, recombination, or due to artifacts such as specific combinations of sample preparation, sequencing technology, consensus calling approaches, and sequencing errors. In order to remove spurious homoplasic sites, a particular worry of this data set because a mix of technologies and methods have been employed by different contributing research groups, these were filtered using a set of parameters and thresholds defined in (van Dorp et al. 2020) to obtain a set of high-confidence homoplasies. Briefly, for each homoplasy, the proportion of isolates with the homoplasy where the nearest neighboring isolate in the phylogeny also carried the homoplasy (pnn) was computed and all homoplasies with pnn <0.1 were excluded. Furthermore, we also excluded homoplasies that were shared in <0.1% of the isolates (>11 isolates). We also required that no isolate had an ambiguous base near the homoplasies (±5 bp). These filters reduced the number of homoplasic sites to 67. The predicted equilibrium frequency of the four nucleotides at all the homoplasic sites (440 mutations), counting each class of mutation only once, is not different from that at nonhomoplasic sites (Euclidean distance method: *P* = 0.61). The filtered (accepted) homoplasies are also not significantly different from nonhomoplasic mutations (Euclidean distance method: *P* = 0.63) or from the rejected ones (Euclidean distance method: *P* = 0.41). We conclude that the accepted set, with each mutation counted once, presents a defendable balance between inclusion and stringency.

### Estimating Dinucleotide Enrichment

For the dinucleotide NpM (e.g., CpG and GpC), we define gene body enrichment (*E*(NM)) as:

E(NM)=p (NM)/[p(N)×p(M)],

where *p*(NM) is the frequency of all dinucleotides within the gene that are NM and *p*(N) and *p*(M) are the frequencies of the mononucleotides within the same gene. We then consider site-specific enrichment, that is, sites 12, 23, or 31 defined by codon position, 31 being a third site and the codon first site of the following codon. Then at sites xy:

$$E\left({\text{NM}}_{\text{xy}}\right)=p\left({\text{NM}}_{\text{xy}}\right)/\left[p\left(N_{x}\right)\times p\left(M_{y}\right)\right],$$

where NM_xy_ is the relevant dinucleotide initiating with *N* at site x.

### Gene Expression

We employ expression data specified by Kim et al. (2020). We used the highest read count for each subgenomic RNAs in Supplementary Table 3 of Kim et al. (2020) and compared log2 normalized read counts to gene G+C content, G+C at third sites, and CpG enrichment. As the authors employed nanopore sequencing, read count does not obviously require gene length normalization. Note that subgenomic RNA measures exclude ORF1a and ORF1b. ORF10 is excluded as no reads were identified. We used the shapiro.test function in R to test log2-transformed read counts for normality.

### RNA Stability

The minimum free energy of mRNA secondary structure was calculated for entire SARS-CoV-2 coding sequences (as defined in Gene Locations), using the hybrid-ss-min (UNAFold) program version 3.8 (Markham and Zuker 2008), with default settings (NA = RNA, *t* = 37). The folding energy of each sequence was then divided by the length of the corresponding sequence, to obtain the per nucleotide mRNA stability measure that was used in downstream calculations.

### Data Compilation of Vertebrate Viruses

Vertebrate virus sequences were retrieved from the Virosaurus database (Virosaurus databases 2020_4.1, Release April 2020, file: Virosaurus90v 2020_4.1) (Gleizes et al. 2020) (accessed May 7, 2020). In this database, complete sequences were clustered at 90% to remove redundancy. As in this database, herpesviridae and poxviridae are split in genes rather than full genomes, complete sequences for these viruses were retrieved from NCBI RefSeq database (Pruitt et al. 2005). The same was also done for segmented viruses to allow calculation of sequence parameters per species. Genome classification was retrieved from ICTV Virus Metadata Repository: version May 1, 2020; MSL35 (Walker et al. 2019). Annotation for replication compartments was assigned according to ICTV (Walker et al. 2019) and ViralZone (Hulo et al. 2011) CpG and UpA enrichment were calculated as above. For virus sequences obtained from the Virosaurus database, the mean was derived to obtain one value per species. For segmented viruses, segments were first concatenated before calculating sequence parameters. Species information and sequence parameters can be found in supplementary table 2, Supplementary Material online.

### Genome Sources

We acknowledge the sources of the genomes that we employed in supplementary table 3 (for SARS-CoV-2), supplementary table 4 (for H1N1), and supplementary table 5 (for Ebola), Supplementary Material online.

## Supplementary Material

Supplementary data are available at *Molecular Biology and Evolution* online.

## Supplementary Material

msaa188_Supplementary_Data
