# Corrigendum to: Evidence for Strong Mutation Bias toward, and Selection against, U Content in SARS-CoV-2: Implications for Vaccine Design

**DOI:** 10.1093/molbev/msab257

**Published:** 2021-09-14

**Authors:** Alan M Rice, Atahualpa Castillo Morales, Alexander T Ho, Christine Mordstein, Stefanie Mühlhausen, Samir Watson, Laura Cano, Bethan Young, Grzegorz Kudla, Laurence D Hurst

*Molecular Biology and Evolution*, Volume 38, Issue 1, January 2021, Pages 67–83, https://doi.org/10.1093/molbev/msaa188

In the originally published version of this manuscript, the Figure 7A that should have been about GpC was a replicate of Figure 3 about CpG. The correct Figure 7A is as given here. The authors apologize for any inconvenience.

