# Supplementary material for: Evidence for Strong Mutation Bias toward, and Selection against, U Content in SARS-CoV-2: Implications for Vaccine Design
Source: Mol Biol Evol. 2020 Jul 20;38(1):67–83. doi: 10.1093/molbev/msaa188 (PMC7454790; doi:10.1093/molbev/msaa188)
Supplement: msaa188_Supplementary_Data [file msaa188_supplementary_data.zip › ST1.pdf]

Ligand binding by antiviral Proteins

	Ligand binding	Sequence Binding preference
MDA5	*long dsRNA*[1–4]	May have preference for AU-rich sequences – currently unclear [1,5,6]
LGP2	*5' triphoshphate ssRNA, dsRNA*[7]	No known preference - binds to termini of ligands[4,7,8]
RIGI	*5'triphosphate ssRNA or short dsRNA*[3,9]	poly (U/UC), poly (A/AG) [10,11]
OAS/RNAse L	*dsRNA (**OAS**)*[13]	AU rich hairpins and short dsRNA [5,6,12]<br>OAS1 activated by NNWWNNNNNNNNNWGN motif and GU wobble bases in dsRNA[14,15]<br>OAS2/3 no known preference
	*dsRNA, ssRNA (**RNase L**)*[16,17]	Upon activation by OAS, cleaves predominantly after UpU and UpA dinucleotides[17,18]
PKR	*dsRNA*[19]	No preference[20]
ILF3	*dsRNA, ssRNA*[21–23]	AREs [24]
TRIM25	*dsRNA, ssRNA*[25]	GC rich sequences[25]
ADAR family	*dsRNA*[26]	No sequence binding preference but editing preference depending on ADAR protein[27]<br>ADARs, A> I RNA editing [28,29].
APOBEC3 family	*DNA/RNA*	APOBEC3A, G>A RNA editing[30], C>U DNA editing, prefers TC dinucleotides[31–33]<br>APOBEC3B, C>U DNA editing[34]. TC dinucleotide preference, primarily targets Retrotransposons[35]<br>APOBEC3C, D, and F, C>U DNA and RNA editing at TC/CT/CC dinucleotides[33,36–38]<br>APOBEC3G, C>U DNA and RNA editing, preference for CCC motif and CC dinucleotides[39,40]<br>APOBEC3H, C>U DNA editing, preference for 5mCpG-C/G and CpG-C/G motifs and TC dinucleotides[33,41,42]
ZAP	*ssRNA*[43,44]	CpG[45]
DDX17	*hairpins on ssRNA*[46]	CA and CT repeats[46]
cGAS	*Cytoplasmic dsDNA*[47]	short region of dsDNA flanked with at least 3 G nucleotides[47]
DAI	*Cytoplasmic DNA* [48]	No preference[49]
IFI16	*Cytoplasmic DNA*[50]	No preference[51]
TLR7/8	*Endosomal ssRNA*[52]	GU, AU or poly U rich ssRNA [52–56]
TLR3	*Endosomal dsRNA*[57]	No preference[57]
TLR9	*Endosomal dsDNA*[58]	Unmethylated CpG rich requences[58]

Supplementary Table 1 References

1. Wu, B. *et al.* Structural basis for dsRNA recognition, filament formation, and antiviral signal activation by MDA5. *Cell* (2013) doi:10.1016/j.cell.2012.11.048.
2. Hornung, V. *et al.* 5′-Triphosphate RNA is the ligand for RIG-I. *Science (80-.).* (2006) doi:10.1126/science.1132505.
3. Kato, H. *et al.* Length-dependent recognition of double-stranded ribonucleic acids by retinoic acid-inducible gene-I and melanoma differentiation-associated gene 5. *J. Exp. Med.* (2008) doi:10.1084/jem.20080091.
4. Uchikawa, E. *et al.* Structural Analysis of dsRNA Binding to Anti-viral Pattern Recognition Receptors LGP2 and MDA5. *Mol. Cell* (2016) doi:10.1016/j.molcel.2016.04.021.
5. Runge, S. *et al.* In Vivo Ligands of MDA5 and RIG-I in Measles Virus-Infected Cells. *PLoS Pathog.* (2014) doi:10.1371/journal.ppat.1004081.
6. Sanchez David, R. Y. *et al.* Comparative analysis of viral RNA signatures on different RIG-I-like receptors. *Elife* (2016) doi:10.7554/eLife.11275.
7. Bamming, D. & Horvath, C. M. Regulation of signal transduction by enzymatically inactive antiviral RNA helicase proteins MDA5, RIG-I, and LGP2. *J. Biol. Chem.* (2009) doi:10.1074/jbc.M807365200.
8. Li, X. *et al.* The RIG-I-like receptor LGP2 recognizes the termini of double-stranded RNA. *J. Biol. Chem.* (2009) doi:10.1074/jbc.M900818200.
9. Jiang, F. *et al.* Structural basis of RNA recognition and activation by innate immune receptor RIG-I. *Nature* (2011) doi:10.1038/nature10537.
10. Schnell, G., Loo, Y. M., Marcotrigiano, J. & Gale, M. Uridine Composition of the Poly-U/UC Tract of HCV RNA Defines Non-Self Recognition by RIG-I. *PLoS Pathog.* (2012) doi:10.1371/journal.ppat.1002839.
11. Uzri, D. & Gehrke, L. Nucleotide Sequences and Modifications That Determine RIG-I/RNA Binding and Signaling Activities. *J. Virol.* (2009) doi:10.1128/jvi.02449-08.
12. Baum, A., Sachidanandam, R. & García-Sastre, A. Preference of RIG-I for short viral RNA molecules in infected cells revealed by next-generation sequencing. *Proc. Natl. Acad. Sci. U. S. A.* (2010) doi:10.1073/pnas.1005077107.
13. Meurs, E. F. *et al.* Constitutive Expression of Human Double-Stranded RNA-Activated p68 Kinase in Murine Cells Mediates Phosphorylation of Eukaryotic Initiation Factor 2 and Partial Resistance to Encephalomyocarditis Virus Growth. *J. Virol.* (1992).
14. Kodym, R., Kodym, E. & Story, M. D. 2′-5′-Oligoadenylate synthetase is activated by a specific RNA sequence motif. *Biochem. Biophys. Res. Commun.* (2009) doi:10.1016/j.bbrc.2009.07.167.
15. Donovan, J., Dufner, M. & Korennykh, A. Structural basis for cytosolic double-stranded RNA surveillance by human oligoadenylate synthetase 1. *Proc. Natl. Acad. Sci. U. S. A.* (2013) doi:10.1073/pnas.1218528110.
16. Floyd-Smith, G., Slattery, E. & Lengyel, P. Interferon action: RNA cleavage pattern of a (2′-5′) oligoadenylate-dependent endonuclease. *Science (80-.).* (1981) doi:10.1126/science.6165080.
17. Wreschner, D. H., McCauley, J. W., Skehel, J. J. & Kerr, I. M. Interferon action - Sequence specificity of the ppp(A2′p)nA-dependent ribonuclease. *Nature* (1981) doi:10.1038/289414a0.
18. Han, Y. *et al.* Structure of human RNase L reveals the basis for regulated RNA decay in the IFN response. *Science (80-.).* (2014) doi:10.1126/science.1249845.
19. Meurs, E. *et al.* Molecular cloning and characterization of the human double-stranded RNA-activated protein kinase induced by interferon. *Cell* (1990) doi:10.1016/0092-8674(90)90374-N.
20. Zheng, X. & Bevilacqua, P. C. Activation of the protein kinase PKR by short double-stranded RNAs with single-stranded tails. *RNA* (2004) doi:10.1261/rna.7150804.

21. Schmidt, T. *et al.* Coordinated Action of Two Double-Stranded RNA Binding Motifs and an RGG Motif Enables Nuclear Factor 90 To Flexibly Target Different RNA Substrates. *Biochemistry* acs.biochem.5b01072 (2016) doi:10.1021/acs.biochem.5b01072.

22. Jayachandran, U., Grey, H. & Cook, A. G. Nuclear factor 90 uses an ADAR2-like binding mode to recognize specific bases in dsRNA. *Nucleic Acids Res.* **44**, 1924–1936 (2015).

23. Patel, R. C. *et al.* DRBP76, a double-stranded RNA-binding nuclear protein, is phosphorylated by the interferon-induced protein kinase, PKR. *J. Biol. Chem.* **274**, 20432–20437 (1999).

24. Kuwano, Y. *et al.* NF90 selectively represses the translation of target mRNAs bearing an AU-rich signature motif. *Nucleic Acids Res.* **38**, 225–238 (2010).

25. Choudhury, N. R. *et al.* RNA-binding activity of TRIM25 is mediated by its PRY/SPRY domain and is required for ubiquitination. *BMC Biol.* (2017) doi:10.1186/s12915-017-0444-9.

26. Mannion, N. M. *et al.* The RNA-Editing Enzyme ADAR1 Controls Innate Immune Responses to RNA. *Cell Rep.* (2014) doi:10.1016/j.celrep.2014.10.041.

27. Eggington, J. M., Greene, T. & Bass, B. L. Predicting sites of ADAR editing in double-stranded RNA. *Nat. Commun.* (2011) doi:10.1038/ncomms1324.

28. Valente, L. & Nishikura, K. ADAR Gene Family and A-to-I RNA Editing: Diverse Roles in Posttranscriptional Gene Regulation. *Progress in Nucleic Acid Research and Molecular Biology* (2005) doi:10.1016/S0079-6603(04)79006-6.

29. Gerber, A. P. & Keller, W. RNA editing by base deamination: More enzymes, more targets, new mysteries. *Trends in Biochemical Sciences* (2001) doi:10.1016/S0968-0004(01)01827-8.

30. Niavarani, A. *et al.* APOBEC3A is implicated in a novel class of G-to-A mRNA editing in WT1 transcripts. *PLoS One* (2015) doi:10.1371/journal.pone.0120089.

31. Suspène, R., Aynaud, M. M., Vartanian, J. P. & Wain-Hobson, S. Efficient Deamination of 5-Methylcytidine and 5-Substituted Cytidine Residues in DNA by Human APOBEC3A Cytidine Deaminase. *PLoS One* (2013) doi:10.1371/journal.pone.0063461.

32. Thielen, B. K. *et al.* Innate immune signaling induces high levels of TC-specific deaminase activity in primary monocyte-derived cells through expression of APOBEC3A isoforms. *J. Biol. Chem.* (2010) doi:10.1074/jbc.M110.102822.

33. McDaniel, Y. Z. *et al.* Deamination hotspots among APOBEC3 family members are defined by both target site sequence context and ssDNA secondary structure. *Nucleic Acids Res.* (2020) doi:10.1093/nar/gkz1164.

34. Burns, M. B. *et al.* APOBEC3B is an enzymatic source of mutation in breast cancer. *Nature* (2013) doi:10.1038/nature11881.

35. Bishop, K. N. *et al.* Cytidine deamination of retroviral DNA by diverse APOBEC proteins. *Curr. Biol.* (2004) doi:10.1016/j.cub.2004.06.057.

36. Yu, X. *et al.* Induction of APOBEC3G Ubiquitination and Degradation by an HIV-1 Vif-Cul5-SCF Complex. *Science (80-.).* (2003) doi:10.1126/science.1089591.

37. Milewska, A. *et al.* APOBEC3-mediated restriction of RNA virus replication. *Sci. Rep.* (2018) doi:10.1038/s41598-018-24448-2.

38. Meier, J. C., Kankowski, S., Krestel, H. & Hetsch, F. RNA editing—systemic relevance and clue to disease mechanisms? *Frontiers in Molecular Neuroscience* (2016) doi:10.3389/fnmol.2016.00124.

39. McDougall, W. M., Okany, C. & Smith, H. C. Deaminase activity on single-stranded DNA (ssDNA) occurs in vitro when APOBEC3G cytidine deaminase forms homotetramers and higher-order complexes. *J. Biol. Chem.* (2011) doi:10.1074/jbc.M111.269506.

40. Chelico, L., Pham, P. & Goodman, M. F. Stochastic properties of processive cytidine DNA deaminases AID and APOBEC3G. in *Philosophical Transactions of the Royal Society B: Biological Sciences* (2009). doi:10.1098/rstb.2008.0195.

41. Hultquist, J. F. *et al.* Human and Rhesus APOBEC3D, APOBEC3F, APOBEC3G, and APOBEC3H Demonstrate a Conserved Capacity To Restrict Vif-Deficient HIV-1. *J. Virol.* (2011) doi:10.1128/jvi.05238-11.

42. Gu, J. *et al.* Biochemical Characterization of APOBEC3H Variants: Implications for Their HIV-1 Restriction Activity and mC Modification. *J. Mol. Biol.* (2016) doi:10.1016/j.jmb.2016.08.012.

43. Guo, X., Carroll, J.-W. N., MacDonald, M. R., Goff, S. P. & Gao, G. The Zinc Finger Antiviral Protein Directly Binds to Specific Viral mRNAs through the CCCH Zinc Finger Motifs. *J. Virol.* (2004) doi:10.1128/jvi.78.23.12781-12787.2004.

44. Luo, X. *et al.* Molecular Mechanism of RNA Recognition by Zinc-Finger Antiviral Protein. *Cell Rep.* (2020) doi:10.1016/j.celrep.2019.11.116.

45. Ficarelli, M. *et al.* CpG Dinucleotides Inhibit HIV-1 Replication through Zinc Finger Antiviral Protein (ZAP)-Dependent and -Independent Mechanisms. *J. Virol.* (2019) doi:10.1128/jvi.01337-19.

46. Moy, R. H. *et al.* Stem-loop recognition by DDX17 facilitates miRNA processing and antiviral defense. *Cell* (2014) doi:10.1016/j.cell.2014.06.023.

47. Herzner, A. M. *et al.* Sequence-specific activation of the DNA sensor cGAS by Y-form DNA structures as found in primary HIV-1 cDNA. *Nat. Immunol.* (2015) doi:10.1038/ni.3267.

48. Takaoka, A. *et al.* DAI (DLM-1/ZBP1) is a cytosolic DNA sensor and an activator of innate immune response. *Nature* (2007) doi:10.1038/nature06013.

49. Wang, Z. C. *et al.* Regulation of innate immune responses by DAI (DLM-1/ZBP1) and other DNA-sensing molecules. *Proc. Natl. Acad. Sci. U. S. A.* (2008) doi:10.1073/pnas.0801295105.

50. Unterholzner, L. *et al.* IFI16 is an innate immune sensor for intracellular DNA. *Nat. Immunol.* (2010) doi:10.1038/ni.1932.

51. Jin, T. *et al.* Structures of the HIN Domain: DNA Complexes Reveal Ligand Binding and Activation Mechanisms of the AIM2 Inflammasome and IFI16 Receptor. *Immunity* (2012) doi:10.1016/j.immuni.2012.02.014.

52. Diebold, S. S., Kaisho, T., Hemmi, H., Akira, S. & Reis E Sousa, C. Innate Antiviral Responses by Means of TLR7-Mediated Recognition of Single-Stranded RNA. *Science (80-.).* (2004) doi:10.1126/science.1093616.

53. Heil, F. *et al.* Species-Specific Recognition of Single-Stranded RNA via Till-like Receptor 7 and 8. *Science (80-.).* (2004) doi:10.1126/science.1093620.

54. Gantier, M. P. *et al.* TLR7 Is Involved in Sequence-Specific Sensing of Single-Stranded RNAs in Human Macrophages. *J. Immunol.* (2008) doi:10.4049/jimmunol.180.4.2117.

55. Zhang, Z. *et al.* Structural Analyses of Toll-like Receptor 7 Reveal Detailed RNA Sequence Specificity and Recognition Mechanism of Agonistic Ligands. *Cell Rep.* (2018) doi:10.1016/j.celrep.2018.11.081.

56. Lund, J. M. *et al.* Recognition of single-stranded RNA viruses by Toll-like receptor 7. *Proc. Natl. Acad. Sci. U. S. A.* (2004) doi:10.1073/pnas.0400937101.

57. Holt, A. C., Medzhitov, R., Flavell, R. A. & Alexopoulou, L. Recognition of double-stranded RNA and activation of NF-kappaB by Toll-like receptor 3. *Nature* (2001).

58. Hemmi, H. *et al.* A Toll-like receptor recognizes bacterial DNA. *Nature* (2000) doi:10.1038/35047123.
