# Supplementary material for: Evidence for Strong Mutation Bias toward, and Selection against, U Content in SARS-CoV-2: Implications for Vaccine Design
Source: Mol Biol Evol. 2020 Jul 20;38(1):67–83. doi: 10.1093/molbev/msaa188 (PMC7454790; doi:10.1093/molbev/msaa188)
Supplement: msaa188_Supplementary_Data [file msaa188_supplementary_data.zip › ST7_attenuation_algorithm.pdf]

Evolutionary-informed approach to attenuate SARS-CoV-2 genes

We propose to attenuate each SARS-CoV-2 gene individually in the direction opposite to its selection pressure for optimum fitness. Specifically, we increase a genes' CpG content as a function of its CpG enrichment and its UpA content as a function of its UpA enrichment. In addition, we seek to increase U content. The more CpG (UpA) depleted a gene is, the higher its likelihood to have its CpG (UpA) content increased; the less CpG (UpA) depleted a gene is, the lesser the likelihood for CpG and UpA increase.

For each gene we generate multiple, independent variants. For every variant we inspect all synonymous sites, one at a time. For each synonymous codon we test for the potential to increase CpG, UpA and U content and score accordingly. Scores for CpG and UpA introduction are calculated as 1 / CpG enrichment score and 1 / UpA enrichment score, respectively. Scores for U introduction are constant and set to 5 (value empirically determined). Should multiple conditions apply, we use the highest score.

If a codon does not increase CpG, UpA or U content we use position-dependent codon occurrences in human one-exon genes (Ensemble data) to score by inverse favourability in human. Underlying functions stem from a matrix of probabilities for the last nucleotide in a codon box (six-fold codon boxes being split into their respective four-fold and two-fold sub-boxes), fitted into curves as a function of position. Inverse scores are then calculated as 1 − score.

Per synonymous site, the scores for synonymous codons are normalised such that they sum up to 1. To select one of the thusly scored synonymous codons we draw a random number *r* in interval [0, 1] and select the codon for which sum of all codon scores seen so far exceeds *r*. Thus, the higher the score the likelier a codon is selected. The codon list is shuffled before codon selection to ensure selection between synonymous codons with same score remains non-deterministic. We set all stop codons to TAA as this is reported to be strongest in human and gives another UpA dinucleotide.

For every generated variant we report its CpG enrichment, UpA enrichment and U frequency. For genes with CpG enrichment ≤ 1 we tag all variants with CpG enrichment, UpA enrichment and U frequency raised above wild-type. For genes with CpG enrichment > 1 we tag all variants with U frequency raised above and CpG enrichment below wild-type.

For every SARS-CoV-2 gene we first generate 1000 variants. Subsequently, we reduce the variant cloud by removing all variants with G+C content too high compared to wild-type. As an upper limit for genes with a CpG enrichment score < 1, we use original GC + original GC * (1 − CpG enrichment score). For genes with CpG enrichment score > 1, we use a limit of its original GC.

At each synonymous site, we consider all synonymous codons. In case of six-fold degenerates, this means that increasing U and A content may not only happen at 3rd codon site but instead, for codons from a six-fold codon box, also at 1st and 2nd codon site. Likewise, introducing a cross-codon UpA dinucleotide (U at 3rd site and A at 1st site of 3' codon) might reduce 3' synonymous codon search space from six codons to two. Switching between sub-boxes for six-fold degenerates is not an issue with CpG as either all or none of the codons of a codon box start with G. Still, both CpG and UpA dinucleotides might be introduced at 1st/ 2nd codon sites, 2nd/ 3rd codon sites or 3rd site/ 1st site of 3' codon.

We avoid introducing changes to the overlapping region of genes ORF7a and ORF7b, as well as the pseudoknot structure in ORF1a/b.

The source code for creating SARS-CoV-2 variants as described above is publicly available at https://github.com/smuehlh/transgenes/tree/v2.3/lib/standalone and is tagged as “v2.3”.

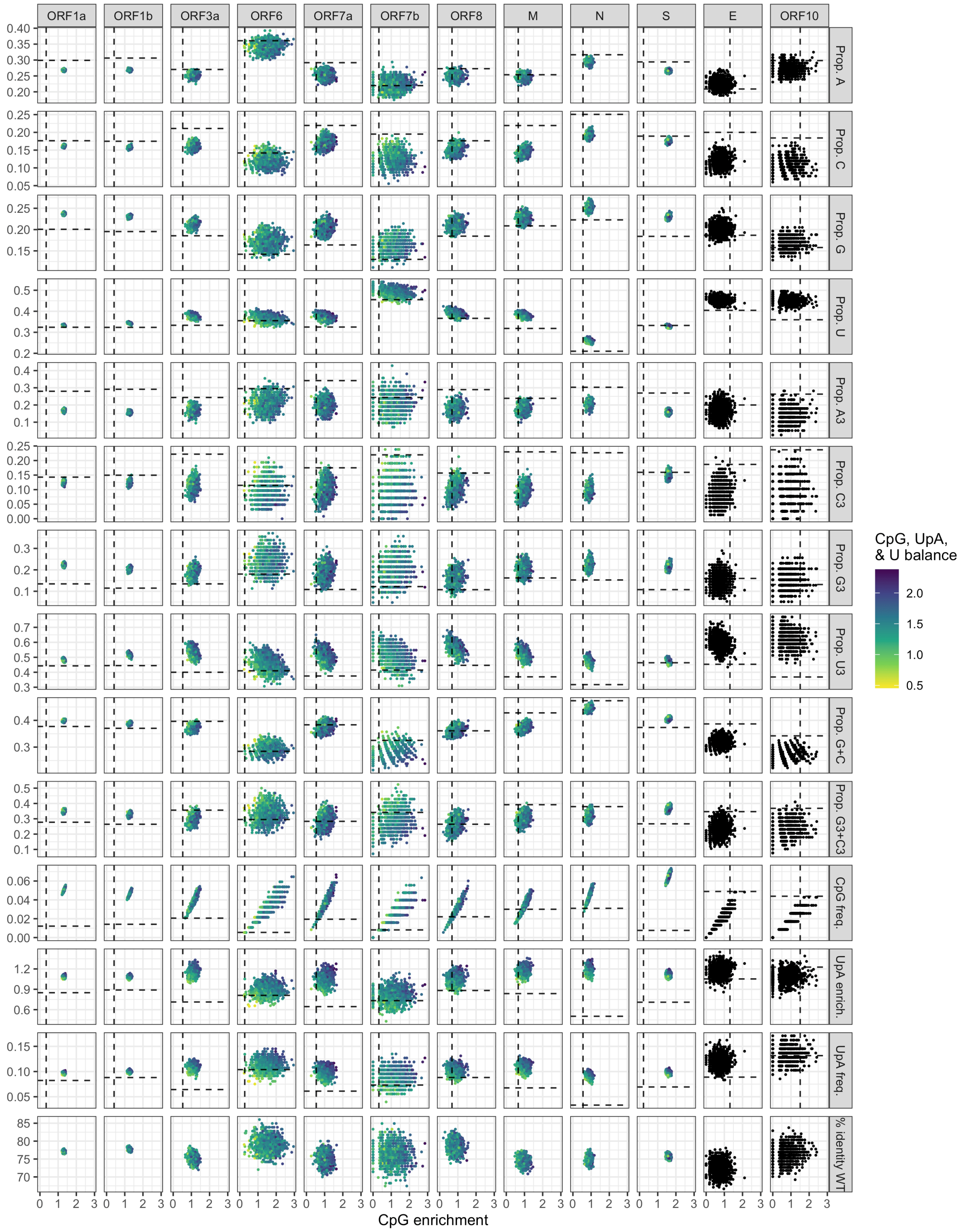

Figure S1 Sequence characteristics of generated variants. Black dashed lines are wild-type observed values for each characteristic. For genes with CpG enrichment score < 1, a “CpG, UpA & U balance” score was calculated by normalising CpG enrichment score, UpA enrichment score, and proportion U, each to between 0 and 1. The three values were summed for each sequence to give a “CpG, UpA & U balance” score. The “best” sequences are those that maximise CpG, UpA, and proportion U, i.e. are *closer to 1 for each characteristic and when summed are closest to 3 (bluer). Prop. – Proportion, Freq. – Frequency, enrich. – enrichment, % identity WT – percentage identity compared to wild-type.*
